# Mesenteric panniculitis with pedal edema in a 33-year-old Pakistani man: a case report and literature review

**DOI:** 10.1186/1752-1947-2-365

**Published:** 2008-12-04

**Authors:** Abdul M Zafar, Muhamad A Rauf, Tabish Chawla, Gule Khanda

**Affiliations:** 1 Department of Radiology, Aga Khan University, Karachi, Pakistan; 2 Department of Surgery, Aga Khan University, Karachi, Pakistan

## Abstract

**Introduction:**

Mesenteric panniculitis is a rare pathology of unknown etiology characterized by inflammation and fibrosis in the mesentery. Its protean clinical and radiological manifestations make it a diagnostic challenge. There is no established treatment available for its management. The clinical outcome is inconsistent, with the prognosis ranging from complete resolution without any treatment to rapid progression culminating in death.

**Case presentation:**

A 33-year-old Pakistani man presented with vague abdominal pain, an ill-defined epigastric mass and bilateral pedal edema. A detailed review of his history and laboratory investigations did not point to any diagnosis. The patient underwent an exploratory laparotomy based on the finding of mesenteric soft-tissue density on computed tomography. The laparotomy did not prove to be of any diagnostic or therapeutic value. Upon review of the pre-operative computed tomographic scan at our institution, a diagnosis of mesenteric panniculitis was made. An acceptable resolution of abdominal pain and pedal edema was attained after a 4-week trial of immunosuppressive therapy. This is the first reported case of mesenteric panniculitis with pedal edema as part of its presentation.

**Conclusion:**

An increased awareness may lead to the development of a less invasive diagnostic approach and optimal treatment for this rarely recognized condition.

## Introduction

Mesenteric panniculitis (MP) is a rare inflammatory and fibrosing disorder of unknown etiology involving adipose tissue of the mesentery [1-5]. Since its first description in 1924, few large series have been reported; the literature is comprised mainly of case reports and studies of small series [3,5,6]. A variety of labels such as mesenteric panniculitis, sclerosing mesenteritis, lipodystrophy and retractile mesenteritis have been applied to the disorder. Conceivably, these represent different points on a spectrum [3-5]. MP presents a diagnostic challenge to physicians, surgeons, radiologists and pathologists alike. This may be attributed to the profusion of differential diagnoses as well as the dearth of literature pertaining to its presentation and diagnosis [5].

We report a case of MP initially presumed to be a liposarcoma. This is the first reported case of MP with pedal edema as part of its presentation. We hope that this report will contribute towards an improved recognition and diagnosis of this rarely diagnosed condition.

## Case presentation

A 33-year-old male Pakistani sailor presented to us with a 1-year history of generalized, vague abdominal pain. The pain was insidious in onset and aggravated by squatting. The patient also complained of bilateral dependent pedal edema that started 1 month after the onset of abdominal symptoms. Furthermore, he reported occasional nausea but did not divulge any history of vomiting, altered bowel habits, malaise, fever or weight loss.

At the onset of these complaints, the patient reported to a military hospital where a palpable epigastric/umbilical mass was discovered. Further evaluation with a computed tomography (CT) scan at the same time demonstrated a fat density mass in the retroperitoneum. A presumptive diagnosis of liposarcoma was made. A laparotomy was performed through a midline incision. Intra-operatively, a vaguely defined thickening was noted in the small bowel mesentery. Complete excision of the mass was limited by its close association with the gut loops. The bowel loops and peritoneal cavity appeared unremarkable. No lymph node enlargement was observed. In the histological evaluation, the excised specimen was described as benign adipose tissue with non-specific inflammatory infiltrates. There was no distortion of the tissue architecture or infiltration of vascular structures. The postoperative recovery was unremarkable.

Nine months after laparotomy, the patient presented to our institution due to persistence of the initial symptoms. A detailed history and review of systems did not provide any further clues. The patient reported no change in abdominal symptoms or pedal edema. His weight had remained stable and he had not experienced any constitutional symptoms. During the initial physical examination, we documented a healed midline laparotomy scar, generalized abdominal tenderness of moderate intensity and a firm, ill-defined mass in the epigastric/umbilical region. Bilateral, pitting pedal edema was also noted. The remainder of the physical examination was unremarkable.

**Figure 1:**
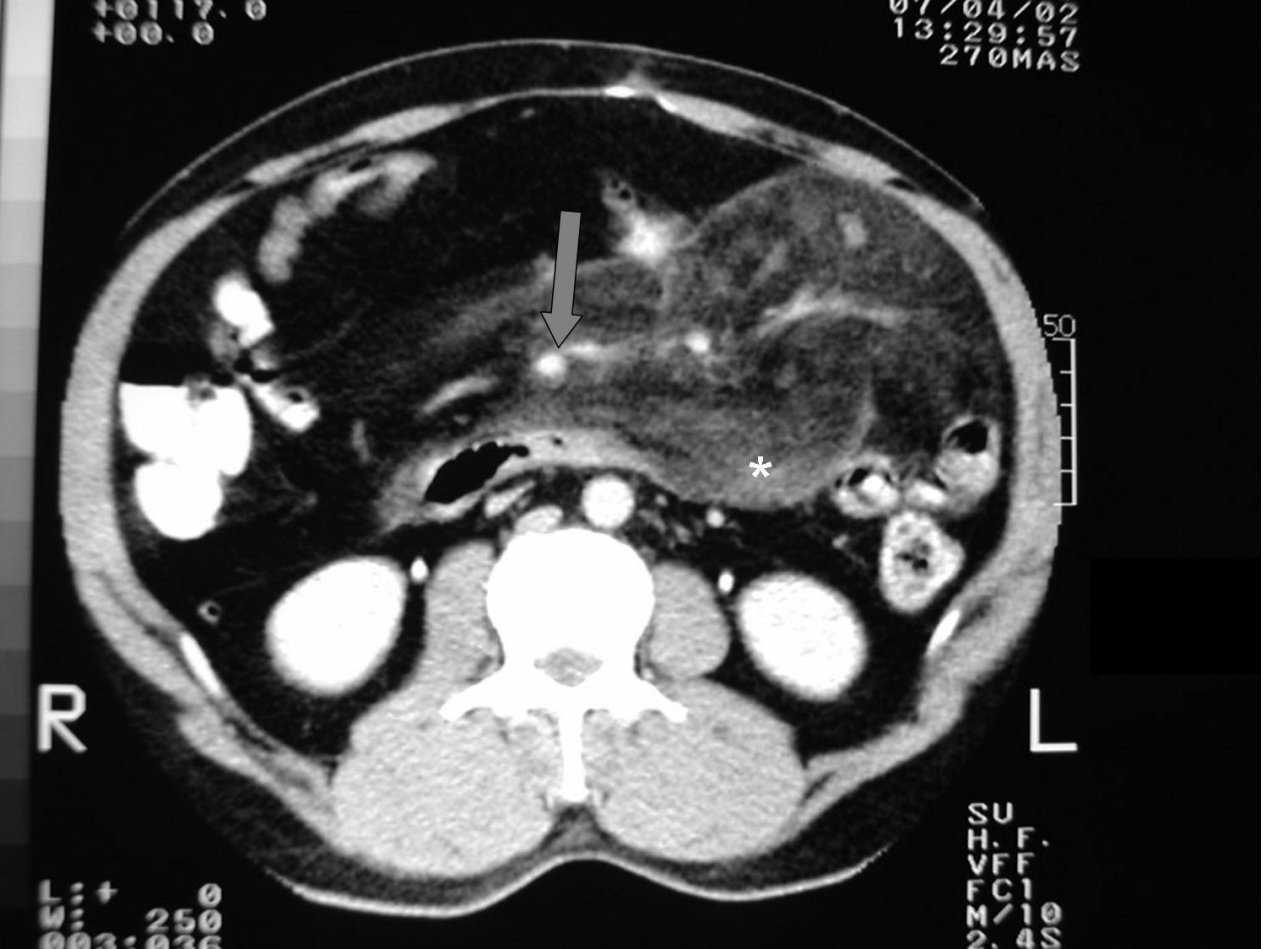
Pre-operative abdominal computed tomography scan of the patient demonstrating characteristic features of mesenteric panniculitis; soft tissue density/mass (*asterisk*) and relative sparing of mesenteric vessels (*grey arrow*).

**Figure 2:**
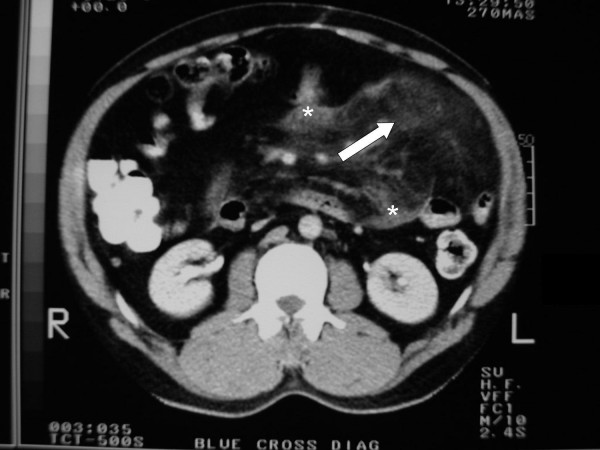
Pre-operative abdominal computed tomography scan of the patient demonstrating characteristic features of mesenteric panniculitis; soft tissue density/mass (*asterisk*) and 'misty mesentery' (*white arrow*).

**Figure 3:**
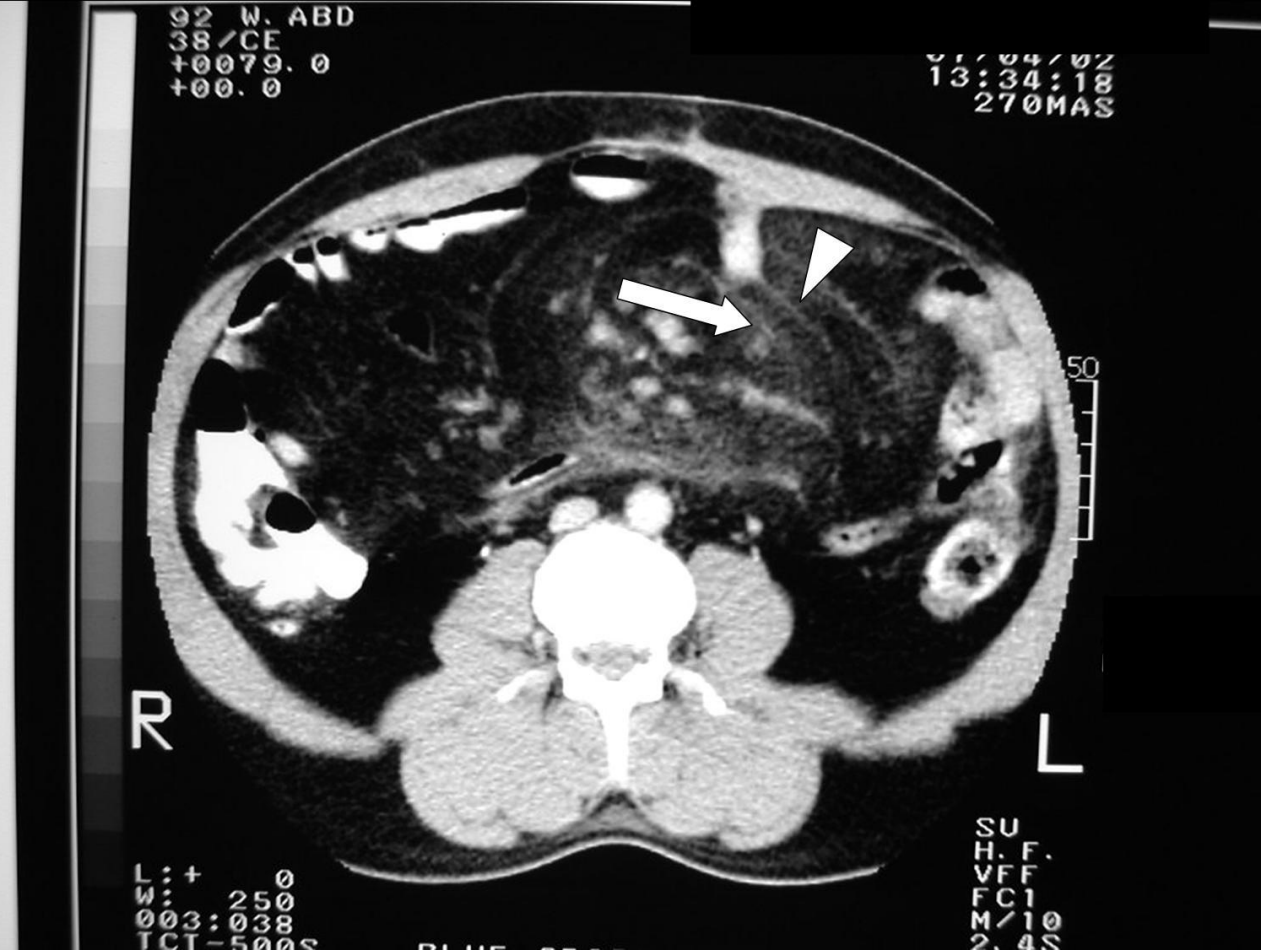
Pre-operative abdominal computed tomography scan of the patient demonstrating characteristic features of mesenteric panniculitis; 'misty mesentry' (*white arrow*) and whorling/stranding of the mesentry (*arrowhead*).

Laboratory studies, including complete blood count, peripheral blood film, erythrocyte sedimentation rate, serum C-reactive protein, amylase, lipase, liver function tests and autoimmune work-up all had negative results. A repeat CT scan was also contemplated but, unfortunately, was not a financially feasible option for our patient. Upon review of the pre-operative CT scan of the abdomen (Figures 1, 2 and 3), diffuse stranding and generalized thickening of the mesentery was noted. A soft tissue density encircling the mesenteric vessels was also identified in the mesentery. There were no signs of bowel obstruction. No abnormality was observed in the lymph nodes, liver or spleen. A retrospective diagnosis of mesenteric panniculitis was established on the basis of CT features, histopathological findings and the benign clinical course.

The patient was started on prednisone 40 mg/day plus azathioprine 50 mg/day and was re-evaluated after 4 weeks. An acceptable symptomatic improvement was observed. The pedal edema resolved completely whereas the pain reduced from five points to two points on a scale of ten points. Azathioprine was discontinued and a tapering dose of steroids was prescribed. The patient declined follow-up.

## Discussion

Mesenteric panniculitis constitutes idiopathic inflammation and fibrosis of the mesentery [1-5]. Mostly, the small-bowel mesentery is affected, although involvement of large bowel mesentery has also been reported [3,4,7,8]. The affected age group can be from 20 to 90 years of age. Men are affected almost twice as commonly as women [3,5,9]. Although the disease is considered idiopathic and benign, it has been associated with malignancies, chronic inflammatory conditions, autoimmune processes, collagen vascular diseases, ischemia, infection and a history of abdominal surgery [3,5,9]. In our case, no associated condition could be identified.

Diagnosing MP presents a challenge. The patient may present with vague abdominal pain, an abdominal mass, altered bowel habits, intestinal obstruction or ascites. MP has been an incidental finding in as many as half of the cases reported [3-6]. Abdominal pain and an abdominal mass, which were also observed in our case, are among the most common manifestations [3,5]. An unprecedented additional complaint in our patient was pedal edema. The symptoms of MP are considered to be secondary to its mechanical effects on the bowel and vascular structures [4,5]. We suspect that the same mechanism may have lead to the development of pedal edema. The link of pedal edema with MP is also suggested by the complete resolution of the pedal edema after treatment.

During the diagnostic work up of MP, extensive biochemical investigations, including immunohistochemical staining, are usually negative. Their value is usually limited to the exclusion of other differentials. Nonetheless, a raised erythrocyte sedimentation rate (ESR) may be seen in a minor proportion of patients [5].

A CT scan may point towards the diagnosis in about half the cases [1,5,6,9]. MP usually appears as a soft tissue density in the base of small bowel mesentery. It may vary from a subtle attenuation to a mass [9]. 'Misty mesentery' (a subtle generalized attenuation in the mesentery) thought to represent chronic inflammation, is a common finding [10]. Calcifications and cystic changes, which probably represent a necrotic process, may also be seen. Fat preservation around mesenteric vessels ('fat ring sign'), an absence of lymph node involvement and the presence of calcifications may help to distinguish MP from some other malignancies [2,9]. Three-dimensional CT and CT angiography may also aid in diagnosis by providing a better perspective of its complex relation to other mesenteric structures [9]. Conversely, the radiographic findings may be indistinguishable from that of other malignant processes in an estimated one-fourth of cases [5].

Histological evaluation is considered imperative for establishing the definitive diagnosis. It may illustrate fat necrosis, chronic inflammation, fibrosis, a combination of any two of these or all of the three [3,5,6]. However, even histological features may closely resemble that of a lymphoma or a desmoplastic reaction [1]. Arguably, the diagnosis can only be reached by a combination of appropriate clinical history, imaging features, intra-operative observations and histological findings [1,5].

Currently, there is no established regimen for the management of MP [5]. The treatment is usually empiric and individualized. Drugs that have been employed include immunosuppressants such as prednisone, colchicine, azathioprine, cyclophosphamide, thalidomide and tamoxifen plus progesterone. Surgery and radiation therapy have also been used for symptomatic relief in certain cases [5,11,12].

The natural course of MP is as inconsistent as its presentation. It may regress, may follow a benign stable course or may progress to death. This variability has been observed in both treated and untreated cases [5,9]. Predominant lipodystrophy usually has a favourable prognosis, whereas primarily fibrotic cases are thought to have a more negative outcome; chronic inflammation lies in the middle of the spectrum [6].

## Conclusion

MP poses a diagnostic challenge owing to its protean presentation, findings and clinical course. An improved understanding may help in increased recognition of this rare, possibly under-recognized, condition. Such efforts may also lead to the development of a less invasive diagnostic approach and improved therapy [5,13]. Careful evaluation of the history and physical examination, together with appropriate imaging, may be helpful in achieving these aims.

## Abbreviations

MP: mesenteric panniculitis; CT: computed tomography; ESR: erythrocyte sedimentation rate.

## Consent

Written informed consent was obtained from the patient for publication of this case report and accompanying images. A copy of the written consent is available for review by the Editor-in-Chief of this journal.

## Competing interests

The authors declare that they have no competing interests.

## Authors' contributions

AMZ, MAR and TC were involved in the direct clinical care of the patient and therapeutic planning, AMZ and GK were involved in the interpretation of diagnostic studies especially the CT scan. All authors contributed equally to the manuscript. All authors have seen and approved the final manuscript and stand responsible for its contents.
